# Acute Effects of Capsaicin on Energy Expenditure and Fat Oxidation in Negative Energy Balance

**DOI:** 10.1371/journal.pone.0067786

**Published:** 2013-07-02

**Authors:** Pilou L. H. R. Janssens, Rick Hursel, Eveline A. P. Martens, Margriet S. Westerterp-Plantenga

**Affiliations:** Department of Human Biology, School for Nutrition, Toxicology and Metabolism (NUTRIM), Maastricht University, Maastricht, The Netherlands; Paris Institute of Technology for Life, Food and Environmental Sciences, France

## Abstract

**Background:**

Addition of capsaicin (CAPS) to the diet has been shown to increase energy expenditure; therefore capsaicin is an interesting target for anti-obesity therapy.

**Aim:**

We investigated the 24 h effects of CAPS on energy expenditure, substrate oxidation and blood pressure during 25% negative energy balance.

**Methods:**

Subjects underwent four 36 h sessions in a respiration chamber for measurements of energy expenditure, substrate oxidation and blood pressure. They received 100% or 75% of their daily energy requirements in the conditions ‘100%CAPS’, ‘100%Control’, ‘75%CAPS’ and ‘75%Control’. CAPS was given at a dose of 2.56 mg (1.03 g of red chili pepper, 39,050 Scoville heat units (SHU)) with every meal.

**Results:**

An induced negative energy balance of 25% was effectively a 20.5% negative energy balance due to adapting mechanisms. Diet-induced thermogenesis (DIT) and resting energy expenditure (REE) at 75%CAPS did not differ from DIT and REE at 100%Control, while at 75%Control these tended to be or were lower than at 100%Control (p = 0.05 and p = 0.02 respectively). Sleeping metabolic rate (SMR) at 75%CAPS did not differ from SMR at 100%CAPS, while SMR at 75%Control was lower than at 100%CAPS (p = 0.04). Fat oxidation at 75%CAPS was higher than at 100%Control (p = 0.03), while with 75%Control it did not differ from 100%Control. Respiratory quotient (RQ) was more decreased at 75%CAPS (p = 0.04) than at 75%Control (p = 0.05) when compared with 100%Control. Blood pressure did not differ between the four conditions.

**Conclusion:**

In an effectively 20.5% negative energy balance, consumption of 2.56 mg capsaicin per meal supports negative energy balance by counteracting the unfavorable negative energy balance effect of decrease in components of energy expenditure. Moreover, consumption of 2.56 mg capsaicin per meal promotes fat oxidation in negative energy balance and does not increase blood pressure significantly.

**Trial Registration:**

Nederlands Trial Register; registration number NTR2944

## Introduction

Obesity is a result of an energy imbalance that develops when energy intake exceeds energy expenditure. Overweight and obesity are the fifth leading risk for global deaths, at least 2.8 million adults die each year as a result of being overweight or obese [1]. Capsaicin, the major pungent principle of red chili pepper, is a thermogenic ingredient which stimulates energy expenditure and contains negligible amounts of energy itself. Therefore, capsaicin may be an interesting target for anti-obesity therapy. Several studies have shown that capsaicin stimulates thermogenesis by increasing the energy expenditure [2], [3], [4], [5], [6]. Furthermore, a decrease in RQ [4] and a beneficial effect of capsaicin on fat oxidation was found [2], [3].

Capsaicin is one of the five naturally occurring capsaicinoids: capsaicin, dihydrocapsaicin, nordihydrocapsaicin, homocapsaicin and homodihydrocapsaicin. The number of Scoville heat units (SHU) indicates the amount of capsaicin present in the pepper. The Scoville scale reflects concentrations of all capsaicinoids, and also expresses the pungency of other capsaicinoids such as nordihydrocapsaicin and dihydrocapsaicin. If a pepper contains 50,000 SHU, this means that its alcoholic extract needs to be diluted 1∶50,000 to be pungent on the human tongue [7]. Red chili pepper can be ingested orally or in capsule form, whereby oral exposure is relatively more effective with respect to thermogenesis [8]. One explanation for effects of capsaicin may be that it produces pain and stimulates thermogenesis caused by stimulating the Transient Receptor Potential Vanilloid receptor 1 (TRPV1) [7], [9]. Another explanation may be that capsaicin causes an increase in catecholamine (epinephrine, norepinephrine and dopamine) secretion and sympathetic nervous system (SNS) activity and consequently, increases blood pressure [4], [10], [11], [12], [13], [14]. Human studies have shown that capsaicin increased the diet-induced thermogenesis [3], [4], [10]. Both human and animal studies investigated the effect of capsaicin after administration of β-adrenergic blockers such as propranolol, and showed that the thermogenic effect of capsaicin is reduced after administration of beta-adrenergic blockers [12], [15]. This implies that the increased thermogenesis by capsaicin is probably based on β-adrenergic stimulation. Whether, in negative energy balance, a reduction in energy expenditure can be prevented by consuming capsaicin, remains to be shown.

Taken together, since capsaicin supplementation adds only negligible amounts of energy to food intake while it increases energy expenditure at least in energy balance, it is of importance to study whether these characteristics may be used as a concept for prevention of the yo-yo effect when entering negative energy balance. Normally, introduction of a negative energy balance by reducing energy intake causes reduction in energy expenditure. We hypothesize that capsaicin supplementation vs. control during negative energy balance counteracts the normal decrease in energy expenditure. Thereby, capsaicin may increase fat oxidation relative to control. The aim of the present study was to investigate the 24 h effects of capsaicin in 25% negative energy balance on energy expenditure and substrate oxidation. We investigated whether the 24 h effects of capsaicin in 25% negative energy balance counteracted the effects of a negative energy balance on energy expenditure and enlarged fat oxidation compared to 100% energy intake without capsaicin.

## Methods

### Subjects

Nineteen healthy Caucasian subjects, aged between 18–50 years, with a body mass index (BMI, kg/m^2^) between 20–30 were recruited for this study. Subjects were recruited by advertisements in local newspapers and on notice boards at Maastricht University. All subjects underwent a medical screening; during this screening subjects underwent anthropometric measurements, and completed questionnaires related to health, smoking behaviour, use of medication, alcohol consumption, physical activity and eating behaviour.

The inclusion criteria, besides an age between 18–50 years and a BMI between 20–30 kg/m^2^, were a good health, non-smoking, not using a more than moderate amount of alcohol (<10 consumptions per week) or caffeine-containing beverages (<2 cups per day). Subjects had to be weight stable (weight change <3kg during the last 6 months), not using medication except for oral contraceptives in women and had to be dietary unrestraint. The Three Factor Eating Questionnaire (TFEQ) was used to determine eating behaviour [16]. Only non-restrained eaters (<10 scores on factor 1), these are persons who are not consciously occupied with food or who are caloric restricted, were selected. Subjects had to be moderately active (<5 hours exercise per week) and used to consuming spicy foods on a regular basis (1–2 days per week, in a low dosage with one meal/day). Pregnant or lactating women were also excluded. Individuals with allergies for the food items used in the study were excluded from participation. Subject sample size was calculated where α was 0.05, β was 0.95 using energy expenditure changes from past papers [11] to calculate the effect size. The sample size was finalized as 14 subjects. The α-level was two-sided.

A written informed consent was obtained from all the participants. The Medical Ethics Committee of the Academic Hospital in Maastricht approved the study. The study was registered as follows: Nederlands Trial Register, registration number NTR2944. The protocol for this trial and supporting CONSORT checklist are available as supporting information; see Checklist S1 and protocol S1.

### Study Protocol

The study had a single-blinded, randomized crossover design with four randomly sequenced experimental conditions. Subjects underwent four 36 h sessions in a respiration chamber for measurements of energy expenditure and substrate oxidation. Two days prior to each session, subjects were provided with a standardized diet to consume at home in order to be fed in energy balance (energy % protein/fat/carbohydrate: 15/30/55), and to receive the same macronutrient proportions as during the respiration chamber experiment. The subjects were instructed to maintain their habitual activity level on the two days before each visit. Subjects were asked to abstain from alcohol consumption on the two days before each visit. Furthermore, they were asked not to drink caffeine after 10∶00 PM on the day before each visit. The four test sessions were conducted at least one week apart for male subjects and four weeks apart for female subjects to prevent possible treatment-induced effects and to take possible effects of menstrual cycle phase on energy intake and energy expenditure in women into account.

### Energy Expenditure

Oxygen consumption and carbon dioxide production were measured in a respiration chamber [17]. The respiration chamber is a 14m^3^ room, which is furnished with a bed, chair, computer, television, radio, telephone, intercom, sink and toilet. The room was ventilated with fresh air at a rate of 70–80 l/min. The ventilation rate was measured with a dry gas meter (type 4; Schlumberger, Dordrecht, Netherlands), and concentrations of oxygen and carbon dioxide were measured with the use of an infrared carbon dioxide analyzer (Uras 3G; Hartmann and Braun, Frankfurt, Germany and 2 paramagnetic oxygen analyzers: Magnos 6G; Hartmann and Braun, and type OA184A; Servomex, Crowborough, United Kingdom). During each 15-min period, 6 samples of outgoing air for each chamber, 1 sample of fresh air, zero gas, and calibration gas were measured. The gas samples to be measured were selected by a computer that also stored and processed the data. 24 h energy expenditure ( = TEE) consists of SMR, DIT and activity-induced energy expenditure (AEE). 36 h energy expenditure and 36 h RQ were measured from 08∶00 h on the day subjects enter the respiration chamber to 20∶00 h on the next day. SMR was defined as the lowest mean energy expenditure measured over 3 consecutive hours between 00∶00 h and 07∶00 h. REE was calculated by plotting energy expenditure against radar output, that are both averaged over 30-min periods. The intercept of the regression line at the lowest radar output represents the energy expenditure in the inactive state ( = REE), which consists of SMR and DIT. DIT was determined by subtracting SMR from REE. AEE was determined by subtracting SMR and DIT from TEE.

### Substrate Oxidation

Carbohydrate, fat, and protein oxidation were calculated from the measurements of oxygen consumption, carbon dioxide production, and urinary nitrogen excretion by using the formula of Carpenter in Brouwer et al [18]. Urine samples were collected from the second void on the day subjects entered the respiration chamber to 20∶00 h on the next day. Samples, 3 per 36 hrs, in order to determine substrate oxidation were collected in containers with 10 ml HCl to prevent nitrogen loss through evaporation. Volume and nitrogen concentration were measured, the latter with a nitrogen analyzer (CHN-O-Rapid; Heraeus, Hanau, Germany). Urinary nitrogen was collected to calculate the RQ and protein balance correctly.

### Blood Pressure

Blood pressure (BP) was measured 15 minutes before each meal; these measurements were taken in sitting position and were made before the meal to avoid variability due to recent (within 2–3 h) food intake [19]. Subjects were instructed to perform triplicate measurements 15 minutes before breakfast, lunch and dinner and were asked to record the mean of these three measurements.

### Body Composition

Body weight was measured using a digital balance and height was measured using a wall-mounted stadiometer. BMI was calculated as body weight (kg) divided by height (m) squared. The deuterium dilution method according to the Maastricht protocol [20] was used to measure total body water (TBW). The subjects were asked to collect a urine sample in the evening just before drinking a deuterium-enriched water solution. After ingestion of this solution, the subject went to bed and no additional consumption was allowed for this period of time. Ten hours after drinking the water solution, another urine sample was collected. The dilution of the deuterium isotope is a measure of the TBW of the subject. Fat mass (FM) was calculated as body weight minus TBW divided by the hydration factor 0.73. Additionally, FM was determined by Bodpod [21] measurements. Fat mass index (FMI) was calculated by FM (kg) divided by height (m) squared. BMI, FM (%) and FMI were used to define body composition. Waist and hip circumference were determined in standing position by a tape measure. Waist circumference was measured at the smallest circumference between rib cage and iliac crest, and hip circumference at the level of the spina iliaca anterior superior. Accordingly, waist-to-hip ratio (WHR) was calculated by dividing waist by hip circumference. Both waist circumference and waist-to-hip ratio were used to define different patterns of body fat distribution.

### Energy Intake and Food Choice

Subjects were fed in energy balance during two days before the test sessions. Subject specific daily energy requirements were calculated based on basal metabolic rate (BMR), which was individually calculated with the equation of Harris-Benedict [22], and multiplied by a physical activity level (PAL) of 1.7. This PAL value of 1.7 represents the average PAL of modern humans, which ranges from 1.5 to 2.0 [23]. In our population in the south of the Netherlands the PAL value of 1.7 is the mean (range 1.6–1.8) of the subjects, with the subject characteristics assessed in the present study. The energy intake level was estimated as such that subjects were not in a positive or a negative energy balance before they entered the respiratory chamber. In the respiration chamber energy requirements were calculated based on a PAL of 1.35. Subject received 100% of their daily energy requirements in the conditions ‘100%Control’ and ‘100%CAPS’ (energy% protein/fat/carbohydrate: 15/30/55), and received 75% of their daily energy requirements in the condition ‘75%Control’ and ‘75%CAPS’ (energy% protein/fat/carbohydrate: 15/30/55). Energy intake was divided over the meals as 20% for breakfast, 40% for lunch, and 40% for dinner. Subjects had to completely finish all drinks and meals within 30 minutes. Negative energy balance in both 75% conditions was calculated by energy intake minus TEE divided by 100% of their daily energy requirements.

### Dosage

Red chili pepper from the Capsicum frutescens L and Capsicum annuum L (McCormick; USA, capsaicin 2484 µ/g, nordihydrocapsaicin 278 µ/g and dihydrocapsaicin 1440 µ/g) was used as source for capsaicin. With respect to the daily dose for capsaicin, the daily value has not been established. The generally recommended daily dose is 1350 mg capsicum with 0.25% capsaicin (40,000 SHU). Capsaicin was given at a dose of 2.56 mg (1.03 g of red chili pepper, 39,050 SHU) with every meal. This dosage was based upon the maximal dosage given in previous studies and in our pre-test [2], [8], [24]. Divided over three meals, a daily total dose of 7.68 mg CAPS was consumed by the subjects.

Preceding the study in the respiration chamber, several tests concerning pungency and spiciness were conducted. In a pre-test several food items were tested in combination with different dosages of red chili pepper. Before the dosage of red chili pepper in the food was determined, the pleasantness of taste, spiciness and pungency of different food products with red chili pepper were assessed to determine whether the amount of red chili pepper was tolerable. Based upon this pre-study the products we chose to offer during the experiment were; breakfast drink original with red chili pepper concentration of 2.0 g/l, pâté with 1.0 g red chili pepper/30 g, tomato juice with 2.0 g red chili pepper/l and pizza containing 2.0 g red chili pepper. The given dose of each component did not exceed the maximum tolerable dose among our subjects.

### Data Analysis

The Statistical Package for the Social Sciences (SPSS) 17.0 was used to perform univariate within-subject analyses. Repeated-measures ANOVA was used to determine possible differences in energy expenditure and its components, substrate oxidation, energy balances and macronutrient balances within-subjects, between the four conditions. Step-down tests were used for pair wise comparisons, post hoc, including Bonferroni corrections. Shapiro-Wilk test was used to determine normality of the variables, these appeared to be normally distributed. All statistical tests are two-sided and differences are considered statistical significant if p<0.05. Values are expressed as means and standard deviations or standard errors.

## Results

### Subject Characteristics

**Figure 1:**
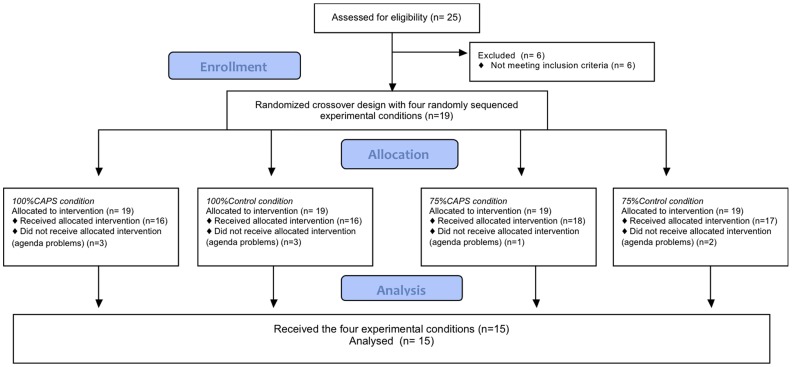
Flow diagram (CONSORT).

Nineteen healthy subjects (nine males, ten females) started the experiments; four subjects dropped-out due to agenda problems. Subjects were used to consuming spicy foods on a regular basis, in general they consumed red chili pepper once per week (0.25–0.5 grams of dried red pepper or 1–2 grams of fresh red pepper). Fifteen subjects (seven female and eight male) completed the four conditions (**Figure 1**); 100% CAPS, 100%Control, 75%CAPS and 75%Control; the subjects had a mean age of 29.7±10.8 y and a mean BMI of 23.3±2.9 kg/m^2^ (**Table 1**).

**Table 1:** Subject characteristics (mean values and standard deviations).

	Male (n = 8)	Female (n = 7)	Total (n = 15)
Age (year)	26.8±8.4	33.0±12.9	29.7±10.8
Height (m)	1.82±0.05	1.65±0.05	1.74±0.10
Body weight (kg)	79.8±10.0	61.2±10.3	71.2±13.7
BMI (kg/m^2^)	24.0±2.6	22.4±3.1	23.3±2.9
FMI (kg/m^2^)	4.4±2.0	6.8±2.0	5.5±2.3
FFMI (kg/m^2^)	19.6±1.5	15.5±1.3	18.6±3.6
WHR	0.80±0.04	0.69±0.04	0.75±0.07
FM (kg)	14.6±6.6	18.8±6.0	16.6±6.5
FFM (kg)	65.3±6.9	42.4±4.8	54.6±13.1
Body fat (%)	17.8±7.1	30.1±4.5	23.6±8.6
TFEQ F1	2.5±2.6	3.1±2.9	2.8±2.7
TFEQ F2	4.9±1.2	5.4±3.4	5.1±2.4
TFEQ F3	4.1±4.3	5.9±3.4	4.9±3.9

BMI: Body mass index; FMI: Fat mass index; FFMI: Fat free mass index; WHR: Waist-to-hip ratio; FM: Fat mass; FFM: Fat free mass; TFEQ: Three Factor Eating Questionnaire; F1, cognitive restraint; F2, disinhibition; F3, hunger. #The TFEQ measures three different factors of human eating behaviour.

### Energy Expenditure

In two conditions subjects received 100% of the daily energy requirements and in the other two conditions they received 75% of the daily energy requirements. Energy balance during 36 h in the 100%CAPS and the 100%Control conditions did not significantly differ from 0 (**Table 2**). During the 75%CAPS and 75%Control conditions negative energy balance was 20.5±1.4% respectively 19.2±1.3%.

**Table 2:** Total energy expenditure, components of energy expenditure, energy intake, substrate oxidation and mean RQ during the four conditions (n = 15).

	100%CAPS	100%Control	75%CAPS	75%Control
EI (MJ/d)	9.09±0.4	9.09±0.4	6.81±0.3**^##^	6.81±0.3**^##^
EB (MJ/d)	0.15±0.2	0.22±0.2	−1.81±0.1**^##^	−1.71±0.1**^##^
TEE (MJ/d)	8.82±0.4	8.75±0.4	8.52±0.4* #	8.41±0.4**#
REE (MJ/d)	7.70±0.4	7.55±0.3	7.49±0.3	7.35±0.3* #
SMR (MJ/d)	6.69±0.3	6.47±0.3	6.45±0.3	6.39±0.3*
DIT (MJ/d)	1.00±0.1	1.09±0.1	1.03±0.1	0.95±0.1#
AEE (MJ/d)	1.12±0.1	1.20±0.1	1.03±0.1	1.06±0.1
Fat oxidation (MJ/d)	1.63±0.2	1.63±0.2	2.38±0.2* #	2.17±0.2*
Carbohydrate oxidation (MJ/d)	5.89±0.2	5.97±0.2	5.03±0.2**^##^	5.18±0.2**^##^
RQ	0.92±0.02	0.92±0.02	0.89±0.02**#	0.90±0.01**#

* p<0.05 compared to 100%CAPS, ** p<0.01 compared to 100%CAPS.

# p<0.05 compared to 100%Control, ^##^ p<0.01 compared to 100%Control.

EI: Energy intake; EB: Energy balance; TEE: Total energy expenditure; REE: Resting energy expenditure; SMR: Sleeping metabolic rate; DIT: Diet-induced thermogenesis; AEE: Activity-induced energy expenditure; RQ: Respiratory quotient.

Total energy expenditure (TEE) in 100%CAPS and 100%Control did not differ significantly. As expected, total energy expenditure (TEE) was higher in the 100% conditions than in the 75% conditions (Table 2); overall an effect on TEE was observed (p = 0.018). With respect to the components of energy expenditure (EE), the following appeared. DIT in the 75%CAPS condition did not differ from DIT in the 100%Control condition, while DIT tended to be lower in 75%Control condition compared with 100%Control condition (p = 0.05). Similarly, REE in the 75%CAPS condition did not differ from REE in the 100%Control condition, while REE was significantly lower in 75%Control condition compared with 100%Control condition (p = 0.02).

Taken together, 75%CAPS did not differ from 100%Control with respect to DIT and REE, while at 75%Control DIT tended to be lower and REE was lower than at 100%Control. Likewise, 75%CAPS did not differ from 100%CAPS regarding SMR, while SMR at 75%Control was lower than SMR at 100%CAPS (p = 0.04).

### Substrate Oxidation

Addition of capsaicin to the meals significantly increased the 24 h fat oxidation in negative energy balance. Fat oxidation in the 75%CAPS condition was significantly higher than fat oxidation in the 100%Control condition (p = 0.03), while at 75%Control fat oxidation did not differ significantly from fat oxidation at 100%Control. Carbohydrate oxidation in 75%CAPS and 75%Control were lower than in 100%Control (p<0.01 and p<0.01 respectively) and than 100%CAPS (p<0.001 and p<0.001 respectively).

**Figure 2:**
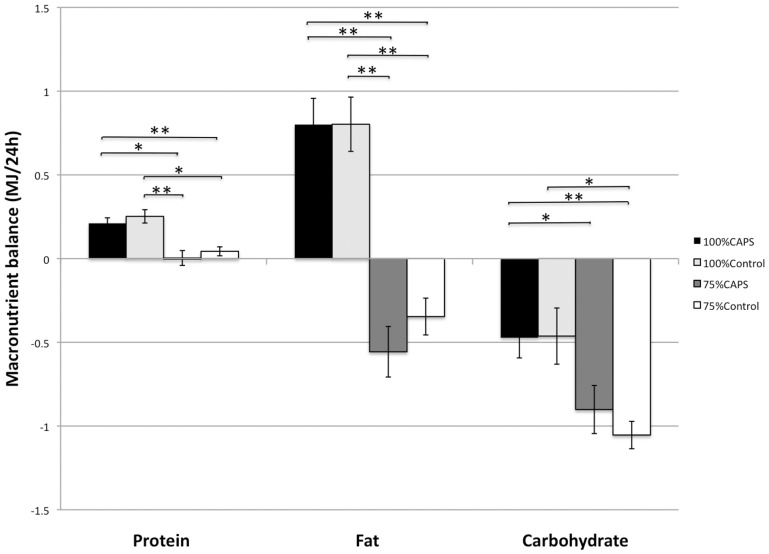
Macronutrient balances for 100%CAPS (black), 100%Control (light grey), 75%CAPS (dark grey) and 75%Control (white) conditions in fifteen subjects (seven female and eight male).

An overall effect of protein balance was observed (p<0.001). Protein balance in the 100%Caps condition was significantly higher than in the 75%CAPS (p<0.05) and in the 75%Control condition (p<0.01); similar findings were present between 100%Control and both 75% conditions (75%CAPS p<0.01, 75%Control p = 0.01). Fat balance was more negative in both 75% conditions and seemed to be more negative in the 75%CAPS than in the 75%Control condition while carbohydrate balance was more negative in the 75%Control (p<0.01) than in the 75%CAPS (p<0.05) condition, vs. 100% conditions. Separate macronutrient balances are shown in **Figure 2**.

### RQ

No significant differences in RQ were seen between the two conditions in energy balance (100%CAPS 0.92±0.02 and 100%Control 0.92±0.02) nor between the two conditions in negative energy balance (75%CAPS 0.89±0.02 and 75%Control 0.90±0.01). RQ was decreased in both 75%CAPS and 75%Control conditions when compared with 100%Control condition. However, RQ was significantly more decreased in the 75%CAPS condition (p = 0.04) than in the 75%Control condition (p = 0.05) when compared with 100%Control (Table 2).

### Blood Pressure

No significant differences were found when systolic and diastolic blood pressure readings were compared between the four conditions **(** **Table 3** **)**.

**Table 3:** Systolic and diastolic blood pressure measurements for the conditions 100%CAPS, 100%Control, 75%CAPS and 75%Control as measured 15 minutes before the meals (n = 15).

		Systolic (mmHg)				Diastolic (mmHg)			
Day	Moment in time	100%CAPS	100%Control	75%CAPS	75%Control	100%CAPS	100%Control	75%CAPS	75%Control
1	Before breakfast	116.1±11.7	117.5±7.2	119.9±10.5	114.9±10.5	72.0±7.9	70.6±6.5	72.9±7.9	69.5±7.8
	Before lunch	118.1±11.3	118.5±11.0	117.7±14.5	113.5±10.2	71.4±8.5	71.8±8.7	70.5±10.1	70.2±7.5
	Before dinner	114.4±11.9	117.6±10.6	120.8±9.9	113.3±12.2	71.9±9.9	70.9±9.0	73.4±8.7	69.3±8.8
2	Before breakfast	116.9±12.9	118.5±12.1	118.5±10.5	116.3±12.3	72.7±7.7	70.7±8.9	69.6±7.2	70.4±10.8
	Before lunch	116.1±11.0	117.5±9.3	119.9±10.4	114.9±13.8	72.0±8.3	70.6±8.1	72.9±9.3	69.5±10.3
	Before dinner	118.1±10.8	118.5±12.2	117.7±11.4	113.5±11.7	71.4±7.4	71.8±7.5	70.5±9.3	70.2±10.1

## Discussion

In the present study we tested the hypothesis that the 24 h effects of capsaicin in 25% negative energy balance would counteract the effects of a negative energy balance on energy expenditure and enlarge fat oxidation compared to 100% energy intake without capsaicin. Therefore we investigated the effects of capsaicin on energy expenditure, substrate oxidation, macronutrient balance and RQ in 100% energy balance and in 75% negative energy balance. Capsaicin was given at a dose of 2.56 mg capsaicin (1.03 g of red chili pepper, 39,050 SHU) with every meal. The induced negative energy balance of 25%, which was obtained by feeding 25% less energy than in energy balance, resulted in a real negative energy balance of 20.5% with CAPS, and of 19.2% with Control.

The strong negative energy balance with capsaicin was due to two main findings. First, DIT and REE in the 75%CAPS condition did not lead to a significant decrease compared with 100%Control, while DIT and REE tended to be or were significantly lower in the 75%Control condition compared with 100%Control. Thus, the effects of capsaicin vs. control in negative energy balance counteracted the effects of the negative energy balance on DIT and REE compared to 100% energy intake without capsaicin. Second, there was a significant increase in fat oxidation when capsaicin was added to the meals in negative energy balance, while there was no significant increase in fat oxidation in the 75%Control condition compared with the 100%Control condition. Since blood pressure did not differ between the four conditions, capsaicin contributed to counteracting effects of negative energy balance without a significant increase in blood pressure. In line with previous studies, that assessed administration of capsaicin in energy balance [3], [4], [5], [6], we expected an increased TEE with 100%CAPS vs. 100%Control. However, this did not reach statistical significance, neither did the components of TEE, namely SMR and DIT. Before, we did observe a larger TEE with 100%CAPS vs. 100%Control [25]; although in the present study differences were observed, lack of statistical significance may be due to the number of subjects included. A long-term study by Lejeune et al. on the effect of 135 mg capsaicin/d on body-weight regain after weight loss found no limiting effect on weight regain after weight loss, yet an increase in thermogenesis and fat oxidation [2]. In this study and another study a similar finding on fat oxidation has been reported, these studies found that capsaicin increased fat oxidation over the long term (3 months) and over the short term (after one breakfast) [2], [3]. The effects of capsaicin on protein oxidation, fat oxidation and carbohydrate oxidation contribute to beneficial effects on body composition and herewith promote an increase in fat free mass and a reduction in FM [26]. Although these beneficial effects on fat oxidation will not guarantee body weight loss or body-weight maintenance, they may counteract a decrease in EE. Yoshioka et al. found that the increase in fat oxidation after capsaicin administration was mainly observed when the meals had a high fat content (energy % protein/fat/carbohydrate: 15/45/40) [3]. In our study the fat content of the meals was normal (energy % protein/fat/carbohydrate: 15/30/55), thus the effect of capsaicin on fat oxidation might have been higher if we would have increased the fat content of the meals. The effects of capsaicin on EE and substrate oxidation do not seem to be acute, but to build up over a few days, since another one-meal study with Caucasians on the acute effect of red chili pepper on satiety, energy expenditure and substrate oxidation found no effect of capsaicin [24]. Given its strong pungency, the long-term use of capsaicin may be limited. A possible solution may be capsinoids. Capsinoids including ‘capsiate’ are non-pungent capsaicinoid analogues. Capsinoids have similar beneficial effects on energy expenditure and substrate oxidation to those of capsaicin [27].

In the present study the subjects received 3.09 g red chili pepper per day (7.68 mg capsaicin); this dosage is relatively low compared to dosages used in studies with Asians [3], [10], [28]. However, there is a difference in maximum tolerable dose of red chili pepper between Asians and Caucasians. This difference in tolerable dose is due to the difference in red chili pepper consumption. Red chili pepper is more common in the food pattern in Asian population. For example, the capsaicin consumption in India is 25–200 mg/day while the average daily consumption in Europe is estimated to be 1.5 mg [29]. Next to studies investigating effects of capsaicin in Caucasians [2], [10], [24], [30], several studies that investigated the effects of capsaicin on appetite and energy intake have been conducted in Asian populations [3], [10], [28].

In summary, the present study shows that the effects of capsaicin vs. control in 25% negative energy balance did prevent the effects of the negative energy balance on DIT and REE compared to 100% energy intake without capsaicin. Moreover, it increased fat oxidation in negative energy balance. The presumed negative energy balance of 25% led to a negative energy balance of 20.5±1.4% when capsaicin was added to the meals and 19.2±1.3% without addition of capsaicin. Since DIT and REE at 75%CAPS were similar as DIT and REE at 100%Control, we conclude that in an effectively 20.5% negative energy balance consumption of 2.56 mg capsaicin per meal supports negative energy balance by counteracting the unfavorable negative energy balance effect of a decrease in components of energy expenditure. Moreover, consumption of 2.56 mg capsaicin per meal promotes fat oxidation in negative energy balance, and does not increase blood pressure significantly.

## Supporting Information

Protocol S1 **Trial Protocol.**
(PDF)

Checklist S1 **CONSORT Checklist.**
(DOC)
