# Supplementary material for: Acute Effects of Capsaicin on Energy Expenditure and Fat Oxidation in Negative Energy Balance
Source: PLoS One. 2013 Jul 2;8(7):e67786. doi: 10.1371/journal.pone.0067786 (PMC3699483; doi:10.1371/journal.pone.0067786)
Supplement: Protocol S1 — Trial Protocol. (PDF) [file pone.0067786.s001.pdf]

C1. Onderzoeksprotocol

RESEARCH PROTOCOL

May 2011

PROTOCOL TITLE

'Acute effects of capsaicin on energy expenditure, fat oxidation and satiety in negative energy balance'

**Protocol ID**	NL 35339
**Short title**	Acute effects of capsaicin
**Version**	3
**Date**	May 2011
**Coordinating investigator**	Prof. dr. M.S. Westerterp-Plantenga<br>Maastricht University, Department of Human Biology, Nutrition and Toxicology Research Institute Maastricht (NUTRIM)<br>P.O. Box 616, 6200 MD Maastricht<br>The Netherlands<br>E-mail: m.westerterp@maastrichtuniversity.nl<br>Phone: +31433881566<br>Fax: +31433670976
**Principal investigators**	Dr. R. Hursel<br>Maastricht University, Department of Human Biology, Nutrition and Toxicology Research Institute Maastricht (NUTRIM)<br>P.O. Box 616, 6200 MD Maastricht<br>The Netherlands<br>E-mail: rick.hursel@maastrichtuniversity.nl<br>Phone: +31433884596<br>Fax: +31433670976<br><br>Msc E.A.P. Martens<br>Maastricht University, Department of Human Biology, Nutrition and Toxicology Research Institute Maastricht (NUTRIM)<br>P.O. Box 616, 6200 MD Maastricht<br>The Netherlands<br>E-mail: eap.martens@maastrichtuniversity.nl<br>Phone: +31433884596<br>Fax: +31433670976
**Legal representative**	Nutrition and Toxicology Research Institute Maastricht (NUTRIM)<br>P.O. Box 616, 6200 MD Maastricht<br>The Netherlands<br>E-mail: mh.grispen@maastrichtuniversity.nl<br>Phone: +31433881476<br>Fax: +31433670286
**Independent physician**	Prof. dr. W.H. Lamers<br>Maastricht University, Department of Anatomy & Embryology, Nutrition and Toxicology Research Institute Maastricht (NUTRIM)<br>P.O. Box 616, 6200 MD Maastricht<br>The Netherlands<br>E-mail: wh.lamers@maastrichtuniversity.nl<br>Phone: +31433881060<br>Fax: +31433884134
**Laboratory sites**	Maastricht University

PROTOCOL SIGNATURE SHEET

Name	Signature	Date
**Legal representative**<br>NUTRIM<br>Sef Janssen		
**Coordinating Investigator**<br>Prof. dr. M.S. Westerterp-Plantenga		

TABLE OF CONTENTS

1. INTRODUCTION AND RATIONALE 9
2. OBJECTIVES 11
3. STUDY DESIGN 12
   3.1. Study design 12
   3.2. Intervention 12
   3.3. Flow chart 13
4. STUDY POPULATION 14
   4.1. Population 14
   4.2. Inclusion criteria 14
   4.3. Exclusion criteria 14
   4.4. Sample size calculation 14
5. TREATMENT OF SUBJECTS 16
   5.1. Investigational product/treatment 16
   5.2. Use of co-intervention 16
6. METHODS 17
   6.1. Study parameters/endpoints 17
      6.1.1. Main study parameter/endpoint 17
      6.1.2. Secondary study parameters/endpoints 17
      6.1.3. Other study parameters 17
   6.2. Randomisation, blinding and treatment allocation 17
   6.3. Study procedures 17
      6.3.1. Body composition 17
      6.3.2. Energy expenditure 18
      6.3.3. Substrate oxidation 18
      6.3.4. Appetite profile 19
      6.3.5. Mood 19
      6.3.6. Energy intake and food choice 19
   6.4. Withdrawal of individual subjects 20
   6.5. Replacement of individual subjects after withdrawal 21
   6.6. Follow-up of subjects withdrawn from treatment 21
7. SAFETY REPORTING 22
   7.1. Section 10 WMO event 22
   7.2. Adverse and serious adverse events 22
   7.3. Follow-up of adverse events 23
8. STATISTICAL ANALYSIS 24
   8.1. Descriptive statistics 24
   8.2. Univariate analysis 24
9. ETHICAL CONSIDERATIONS 25
   9.1. Regulation statement 25
   9.2. Recruitment and consent 25
   9.3. Benefits and risks assessment, group relatedness 25
   9.4. Compensation for injury 27
   9.5. Incentives 27

10. ADMINISTRATIVE ASPECTS AND PUBLICATION 28
10.1. Handling and storage of data and documents 28
10.2. Amendments 28
10.3. Annual progress report 28
10.4. End of study report 28
10.5. Public disclosure and publication policy 28
11. REFERENCES 29

LIST OF ABBREVIATIONS AND RELEVANT DEFINITIONS

ABR	General Assessment and Registration form, the application form that is required for submission to the accredited Ethics Committee (in Dutch: Algemene Beoordeling en Registratie)
AEE	Activity-induced energy expenditure
AR	Adverse Reaction
BMI	Body mass index
CA	Competent Authority
CAPS	Capsaicin
CCMO	Central Committee on Research Involving Human Subjects (in Dutch: Centrale Commissie Mensgebonden Onderzoek)
CV	Curriculum Vitae
DHC	Dihydrocapsaicin
DIT	Diet-induced thermogenesis
DSMB	Data Safety Monitoring Board
EE	Energy expenditure
En%	Energy percent
EU	European Union
EudraCT	European drug regulatory affairs Clinical Trials
FFM	Fat free mass
FM	Fat mass
FMI	Fat mass index
GCP	Good Clinical Practice
IB	Investigator's Brochure
IC	Informed Consent
IMP	Investigational Medicinal Product
IMPD	Investigational Medicinal Product Dossier
METC	Medical Research Ethics Committee (MREC) (in Dutch: Medisch Ethische Toetsing Commissie)
NDC	Nordihydrocapsaicin
PA	Physical activity
POMS	Profile of Mood States
RMR	Resting metabolic rate
RQ	Respiratory quotient
(S)AE	(Serious) Adverse Event

SEM	Standard error of the mean
SHU	Scoville heat units
SMR	Sleeping metabolic rate
SO	Substrate oxidation
SPC	Summary of Product Characteristics (in Dutch: officiële productinfomatie IB1-tekst)
Sponsor	The party that commissions the organisation or performance of the research, for example a pharmaceutical company, academic hospital, scientific organisation or investigator. A party that provides funding for a study but does not commission it is not regarded as the sponsor, but referred to as a subsidising party.
STAI	State-trait anxiety inventory
SUSAR	Suspected Unexpected Serious Adverse Reaction
TBW	Total body water
VAS	Visual analogue scale
WHR	Waist-to-hip ratio
Wbp	Personal Data Protection Act (in Dutch: Wet bescherming persoonsgevens)
WMO	Medical Research Involving Human Subjects Act (in Dutch: Wet Medisch-wetenschappelijk Onderzoek met Mensen

SUMMARY

Rationale: Red pepper might prevent reduction of energy expenditure and elevation of hunger as a result of energy intake restriction.

Objective: To investigate the acute effects of capsaicin on energy expenditure, substrate oxidation, appetite profile and *ad libitum* energy intake during negative energy balance.

Study design: The study will be conducted in a crossover design with four randomly sequenced conditions. Energy expenditure and substrate oxidation will be continuously monitored for 36h. Appetite profile will be measured hourly. *Ad libitum* energy intake will be measured during the last meal.

Study population: Sixteen healthy subjects (8 males and 8 females) with BMI between 20-30 kg/m^2 and aged between 18-50 years will be included in the study. All subjects will be non-smoking, weight stable, dietary unrestraint, and at most moderate alcohol and caffeine consumers. Subject will be free of medication except for oral contraceptives use in women.

Intervention: Subjects will stay for each of the four conditions in a respiration chamber for 36h, twice receiving an energy-balanced (100 En%) diet and twice an energy-restricted (75 En%) diet, both with and without added capsaicin (100%control, 75%control, 100%CAPS, 75%CAPS). An *ad libitum* meal will be served at the end of the second day.

Main study parameters/endpoints: Energy expenditure, substrate oxidation, appetite profile and *ad libitum* energy intake.

Nature and extent of the burden and risks associated with participation:

The study does not include any major risk for the subjects. Anthropometric and body composition measurements, performed during the screening, will not be invasive for the subjects. Deuterium dilution has been shown to be a safe method for determining total body water. Furthermore, the registration of oxygen consumption and carbon dioxide production in the respiration chamber will be performed during an unconscious process. The air in the chamber is continuously regulated.

Urine sampling will be done in urine bottles added with diluted HCl, which might pose a risk for the subjects. However, subjects will be carefully instructed how to handle the bottles to reduce these risks.

Additionally, there are no risks for the subject in consuming any of the provided meals, because people with certain food allergies are excluded for participation and all meals are composed of regular food items available in normal Dutch supermarkets. The addition of capsaicin to the meals will not form any health risk. Capsaicin is a natural product, which is safe in the given dose that will not exceed the maximum recommended daily dose.

Benefit: This study does not have any benefits for the subjects themselves, but will give possible new knowledge for the treatment of obesity.

1. INTRODUCTION AND RATIONALE

Obesity is associated with an increased risk for chronic diseases including type 2 diabetes, cancer, and cardiovascular diseases (1). The development of obesity results from a chronic energy imbalance, with energy intake exceeding energy expenditure. Both decreasing energy intake and increasing energy expenditure may result in weight loss. Modest weight loss (5–10% of body weight) is associated with clinical improvements in insulin sensitivity and fasting glucose levels, thereby reducing the risk for the development of type 2 diabetes (2). Furthermore, reductions in dyslipidemia and hypertension as a result of weight loss reduce the risks for cardiovascular diseases (3).

Under normal circumstances, body weight is very tightly regulated. After a period of energy restriction and weight loss, the body will respond using several mechanisms as increasing feelings of hunger and decreasing energy expenditure to regain its initial body weight (4). These counteractive mechanisms make adherence to weight-loss diets difficult and uncomfortable (5). Therefore, it is of importance to design weight-loss diets that tackle the undesirable physiological responses of the body. Several food components have been studied for their effect on appetite related feelings and thermogenesis. For instance capsaicin, the pungent principle of red pepper, has been reported to increase energy expenditure and diet-induced thermogenesis and to imply a shift in substrate oxidation from carbohydrate to fat oxidation (6-11). For weight-reduction strategies it is necessary to know whether these effects of capsaicin are still present during a moderate energy deficit. In short-term human studies, addition of red pepper to experimental meals significantly increased diet-induced thermogenesis and lipid oxidation (6, 11). In Caucasian male subjects, using a respiration chamber design, red pepper and caffeine consumption was observed to significantly increase 24h energy expenditure (7). Furthermore, in a long-term study, a relatively more sustained fat oxidation in the capsaicin group was observed compared with the placebo group (8). The increase in diet-induced thermogenesis is probably caused by enhancing catecholamine secretion from the adrenal medulla as observed in rats, mainly through activation of the central nervous system (12-17). This is probably based on β-adrenergic stimulation, since both animal and human studies showed that the increase in thermogenesis is abolished after administration of β-adrenergic blockers such as propranolol. Furthermore, the presence of a functional capsaicin-like vanilloid receptor, the Transient Receptor Potential Vanilloid receptor 1 (TRPV1), has been observed in the rats. This vanilloid receptor is expressed in sensory neurons, the brain and various non-neuronal tissues and might mediate oxygen uptake, and thus thermogenesis (12-17).

Capsaicin has also been reported to increase satiety and to decrease appetite and *ad libitum* food intake (7, 10, 18-20). The addition of capsaicin to a test meal was found to reduce food consumption during a subsequent ad libitum meal. A significant decrease in energy intake

was observed after offering capsaicin as an appetizer (18). Red pepper supplementation combined with caffeine significantly reduced the daily ad libitum energy intake (7). In a study of Westerterp-Plantenga et al., average daily energy intake over two days was reduced when capsaicin was offered in tomato juice or in capsules, compared to the control condition (19). Reinbach et al. found that one-day exposure to capsaicin increased satiety during negative energy balance (21). It is necessary to know whether during negative energy balance energy expenditure and fat oxidation are still stimulated more after capsaicin supplementation than in a control situation in the same negative energy balance. The present study aims to investigate the 36h effects of capsaicin on energy expenditure, substrate oxidation and appetite profile, and *ad libitum* energy intake during 25% negative energy balance. It will be investigated whether the 36h effects of capsaicin in 25% negative energy balance prevent the effects of the negative energy balance on energy expenditure, substrate oxidation, appetite profile and *ad libitum* energy intake compared to 100% energy intake without capsaicin.

2. OBJECTIVES

The primary objective of this study is to investigate whether the 36h effects of capsaicin in 25% negative energy balance prevent the effects of the negative energy balance on energy expenditure, substrate oxidation, appetite profile and *ad libitum* energy intake compared to 100% energy intake without capsaicin (75%CAPS vs. 100%Control).
Because of the cross-over design of this study, it needs to be assessed whether, and to which extend, 75% energy intake without capsaicin decreases energy expenditure and satiety compared to 100% energy intake without capsaicin (75%Control vs. 100%Control). In this way, this study will assess the effectiveness of capsaicin as bioactive ingredient during negative energy balance.
The primary objective implies that 100% energy intake with capsaicin increases energy expenditure, fat oxidation and satiety compared to 100% energy intake without capsaicin (100%CAPS vs. 100%Control). Previous studies have shown comparable effects of capsaicin supplementation, but different subject groups and supplementation protocols were used. This study needs to determine whether capsaicin supplementation is effective over 36h in this specific study setting.
Negative energy balance causes a shift in substrate oxidation, leading to a higher fat oxidation compared to energy balance conditions. This study will test the hypothesis that the increase in fat oxidation will be larger in the 75% energy intake with capsaicin condition compared with 75% energy intake without capsaicin condition (75%CAPS vs. 75%Control).

Four conditions will be applied in order to completely answer the research questions:
- 100% energy balance without capsaicin (100%Control)
- 75% energy balance without capsaicin (75%Control)
- 100% energy balance with capsaicin (100%CAPS)
- 75% energy balance with capsaicin (75%CAPS)

3. STUDY DESIGN

3.1. Study design

The study will be conducted in a double-blind crossover design with four randomly sequenced experimental conditions. This 2x2 latin square design will be applied to give a complete picture of the combined effects of capsaicin supplementation and energy balance.

3.2. Intervention

Subjects will stay for four conditions in a respiration chamber for 36h, twice receiving an energy-balanced (100 En%) diet and twice an energy-restricted (75 En%) diet, both with and without added capsaicin (100%Control, 75%Control, 100%CAPS, 75%CAPS). The four test sessions will be conducted four weeks apart to prevent possible treatment-induced effects and to take eventual effects of menstrual cycle phase on energy intake and energy expenditure in women into account.
Two days prior to each test session subjects will be provided with a diet at home, in energy balance and with the same macronutrient composition as they will receive during the test sessions. On each of the four test days subjects arrive at the University after an overnight fast at 8:00h, whereafter they enter the respiration chamber at 8:30h. Energy expenditure, substrate oxidation and activity level will be continuously measured from 8:30h to 20:30h on the next day. Breakfast will be provided at 8:30h and will consist of wholegrain bread with low-fat margarine, liver paste and strawberry jam, and chocolate milk. At 13:30h subjects will receive lunch, which will consist of wholegrain bread with low-fat margarine, liver paste and meatloaf, ginger cake, low-fat milk, apple and raisin biscuits. Dinner at 18:30h will consist of mixed salad with dressing, vegetable lasagne, low-fat yoghurt and low-fat milk. As part of each meal subjects will consume tomato juice (100%Control, 75%Control conditions) or tomato juice with added capsaicin (100%CAPS, 75%CAPS conditions). Subjects will be instructed to go to sleep around 23:30h. The next morning, subjects will be waked at 7:30h. Thereafter, the same protocol as during the previous day will be followed, until dinner. At 18:30h the subjects will receive an *ad libitum* meal, consisting of iceberg lettuce with cucumber, tomato and dressing, macaroni Bolognese, a dessert and orange juice.

Appetite profile will be measured using anchored 100-mm visual analogue scales (VAS). These questionnaires will be completed every waking hour, and before and after every meal on feelings of hunger, fullness, appetite, satiety, thirst, prospective food consumption, and desire to eat. Mood and anxiety levels will be measured by Profile of Mood State (POMS) (22) and State-trait Anxiety Inventory (STAI) (23) questionnaires at the same time points.

Urine samples are collected from the second void on the day subjects enter the respiration chamber to 20:30h on the next day. A total of 4 samples will be collected in containers in order to determine the circadian rhythm of substrate oxidation.

3.3. Flow chart

Day 1

**Time**	**8:00**	**8:30**	**9:00**	**10:00**	**11:00**	**12:00**	**13:00**	**13:30**	**14:00**	**15:00**	**16:00**	**17:00**	**18:00**	**18:30**	**19:00**	**20:00**	**20:30**	**21:00**	**22:00**	**23:00**
Arrival	X																			
EE		>>																		
PA		>>																		
Meal		X						X						X						
Urine		X															X			
VAS		X	X	X	X	X	X	X	X	X	X	X	X	X	X	X		X	X	X
POMS		X	X	X	X	X	X	X	X	X	X	X	X	X	X	X		X	X	X
STAI		X	X	X	X	X	X	X	X	X	X	X	X	X	X	X		X	X	X

Day 2

**Time**	**7:30**	**8:00**	**8:30**	**9:00**	**10:00**	**11:00**	**12:00**	**13:00**	**13:30**	**14:00**	**15:00**	**16:00**	**17:00**	**18:00**	**18:30**	**19:00**	**20:00**	**20:30**
Wake up	X																	
EE	>>																	
PA	>>																	
Meal			X						X						X			
Urine			X															X
VAS		X	X	X	X	X	X	X	X	X	X	X	X	X	X	X	X	
POMS		X	X	X	X	X	X	X	X	X	X	X	X	X	X	X	X	
STAI		X	X	X	X	X	X	X	X	X	X	X	X	X	X	X	X	

4. STUDY POPULATION

4.1. Population

Subjects from the student population will be recruited by advertisements on notice boards at the Maastricht University. Healthy, non-smoking, males and females who are normal weight or overweight (BMI) and aged between 18-50 yrs will be recruited for an initial screening, whereafter a total of 16 subjects (8 males and 8 females) will be included in the study. Because of metabolic disturbances, as the reduced ability to oxidize fat, during the development of obesity are expected to influence the effectiveness of capsaicin treatment, both normal weight and overweight subjects will be included in the study to make comparison between these groups possible (24). During the screening, subjects will undergo anthropometric and body composition measurements, and will complete questionnaires related to health, smoking behavior, use of medication, alcohol consumption, physical activity, eating behavior, mood and anxiety.

4.2. Inclusion criteria

A total of 16 subjects (8 males and 8 females) with BMI 20-30 kg/m^2 and aged between 18-50 years will be included in the study. All subjects will be healthy, non-smoking, not using a more than moderate amount of alcohol (> 10 consumptions/wk) or caffeine-containing beverages (> 2 cups/d), being weight stable (weight change < 3kg during the last 6 months), dietary unrestraint and not using medication or supplements except for oral contraceptives in women.

4.3. Exclusion criteria

Subjects will be excluded if they are not healthy, smoking, using a more than moderate amount of alcohol or caffeine-containing beverages, not being weight stable, dietary restraint, using medication or supplements except for oral contraceptives in women, or if they do not meet the criteria for BMI and age. Pregnant and lactating women, and subjects with allergies for the used food items will also be excluded from participation.

4.4. Sample size calculation

The primary endpoint of this study is the change in 36h energy expenditure.

For the sample size calculation the following formula was used:

$$N = 2 + (Z_{1-\beta} + Z_{1-\alpha/2})^2 \times \sigma^2 / (\mu_{75\%CAPS} - \mu_{75\%CONTROL})^2$$

Herein the β was set at 0.10, which results from a power of 0.9. Consequently, $Z_{0.9} = 1.28$. For the α a value of 0.05 was chosen, which resulted in $Z_{0.975} = 1.96$.

It is hypothesized that the 25 En% restriction CAPS diet will show a relatively larger 36h energy expenditure compared with the 25 En% restriction control diet. A study by Yoshioka et al. showed that a combination of red pepper and caffeine significantly increased 24h energy expenditure compared to a control condition (10190±1550 vs. 9870±1490, p<.05) (7). This corresponds to an increase in 24h energy expenditure of 320 kJ/24h. Therefore, the smallest change in 36h energy expenditure ($\mu_{75\%CAPS} - \mu_{75\%CONTROL}$) was set at 1.5*320 = 480 kJ/36h. The standard deviation was set at 550 kJ/36h, based on several studies performed in the same setting to measure energy expenditure with a respiration chamber as will be used in the current study (25, 26).

The calculated sample size will be:
$N = 2 + (1.28 + 1.96)^2 \times 550^2 / 480^2 = 14$ subjects
Taking a drop-out of 10% into account, 16 subjects will be included in the experiment.

5. TREATMENT OF SUBJECTS

5.1. Investigational product/treatment

Red pepper from the *Capsicum frutescens L and Capsicum annuum L* (McCormick; origin, India; sample ID, EC88746100) will be used as source for capsaicin. This specific form of red pepper has previously been used in a study of Ludy et al, investigating the effects of red pepper on thermogenesis and appetite (11). The product contains nordihydrocapsaicin (NDC; 930 Scoville heat units (SHU)), CAPS (26380 SHU) and dihydrocapsaicin (DHC; 11740 SHU), making a total amount of CAPS of 39050 SHU. Capsaicin will be given at a dose of 80.000 SHU, corresponding to an amount of 5.12 mg capsaicin (2.05 g of red pepper), with every meal. This dosage is based upon the maximal dosage given in previous studies (8, 19, 20). Divided over three meals, a daily total dose of 15.36 mg CAPS will be consumed by the subjects. Subjects will receive the capsaicin for two subsequent days. Because it is suggested that the maximum tolerable dose (about 6.0 g/L) of red pepper is necessary to have a suppressive effect of red pepper on fat intake (27) and that hedonics may be reduced when the concentration of red pepper is higher than 6.0 g/L of red pepper (10), the tomato juice with capsaicin that we chose to offer during this experiment contains 6.0 g/L of red pepper. Per meal subjects will consume 2.05 g of red pepper dissolved in 250 mL tomato juice.

5.2. Use of co-intervention

Two days prior to each test day, subject will be provided with a diet in energy balance, to be consumed at home. All food items will be bought in a normal Dutch supermarket.

The diet consumed at home will consist of:

- Breakfast: wholegrain bread with low-fat margarine, liver paste and strawberry jam, and chocolate milk
- Lunch: wholegrain bread with low-fat margarine, liver paste and meatloaf, ginger cake, low-fat milk, apple and raisin biscuits
- Diner: kale with sausage and jus, fruit cocktail, vanilla custard and orange juice

The diet consumed during the test sessions will consist of:

- Breakfast: tomato juice, wholegrain bread with low-fat margarine, liver paste and strawberry jam, and chocolate milk
- Lunch: tomato juice, wholegrain bread with low-fat margarine, liver paste and meatloaf, ginger cake, low-fat milk, apple and raisin biscuits
- Diner: tomato juice, mixed salad with dressing, vegetable lasagne, low-fat yoghurt and orange juice

6. METHODS

6.1. Study parameters/endpoints

6.1.1. Main study parameter/endpoint

The primary endpoint of this study is the change in 36h energy expenditure.

6.1.2. Secondary study parameters/endpoints

- Substrate oxidation (fat and carbohydrates)
- Sleeping metabolic rate (SMR)
- Diet-induced thermogenesis (DIT)
- Activity induced thermogenesis (AEE)
- Substrate oxidation: carbohydrate, protein, respiratory quotient (RQ)
- Feelings of hunger and satiety: VAS for hunger, fullness, appetite, satiety, thirst, prospective food consumption, desire to eat
- *Ad libitum* energy intake
- Mood: POMS score
- Anxiety: STAI score

6.1.3. Other study parameters

- Age
- Body composition: body weight, BMI, body fat percentage, fat mass index (FMI)
- Body fat distribution: waist circumference, waist-to-hip ratio (WHR)

6.2. Randomisation, blinding and treatment allocation

The study will be conducted in a double-blinded crossover design with four randomly sequenced experimental conditions.

6.3. Study procedures

6.3.1. Body composition

Body weight will be measured using a digital balance and height by a wall-mounted stadiometer. BMI will be calculated as body weight (kg) divided by height (m) squared. The deuterium dilution method according to the Maastricht protocol will be used to determine total body water (TBW) (28, 29). The subjects will be asked to collect a urine sample in the evening just before drinking a deuterium-enriched water solution. After ingestion of this solution, the subject will go to bed and no additional consumption is allowed for that period. Ten hours after drinking the water solution, another urine sample will be collected. The dilution of the deuterium isotope is a measure of the TBW of the subject. Fat mass (FM) will be calculated as body weight minus TBW divided by the hydration factor 0.73 (30).

Additionally, fat mass will be determined by Bodpod measurements. Fat mass index will be calculated by fat mass (kg) divided by height (m) squared. BMI, FM (%) and FMI will be used to define body composition. Waist and hip circumference will be determined in standing position by a tape measure. Waist circumference will be measured at the smallest circumference between rib cage and iliac crest, and hip circumference at the level of the spina iliaca anterior superior. Accordingly, WHR will be calculated by dividing waist by hip circumference. Both waist circumference and waist-to-hip ratio will be used to define different patterns of body fat distribution.

6.3.2. Energy expenditure

Oxygen consumption and carbon dioxide production will measured in the respiration chamber (31). The respiration chamber is a 14-m^3 room furnished with a bed, chair, computer, television, DVD-player, radio, telephone, intercom, sink, and toilet. The room will be ventilated with fresh air at a rate of 70–80 L/min. The ventilation rate will be measured with a dry gas meter (type 4; Schlumberger, Dordrecht, Netherlands), and concentrations of oxygen and carbon dioxide will be measured with the use of an infrared carbon dioxide analyzer (Uras 3G; Hartmann and Braun, Frankfurt, Germany and 2 paramagnetic oxygen analyzers: Magnos 6G; Hartmann and Braun, and type OA184A; Servomex, Crowborough, United Kingdom). During each 15-min period, 6 samples of outgoing air for each chamber, 1 sample of fresh air, zero gas, and calibration gas will be measured. The gas samples to be measured are selected by a computer that will also store and process the data.

24h energy expenditure consists of SMR, DIT and AEE. 36h energy expenditure and 36h RQ will be measured from 08:30h on the day subjects enter the respiration chamber to 20:30h on the next day. SMR is defined as the lowest mean energy expenditure measured over 3 consecutive hours between 00:00h and 07:00h. Resting metabolic rate (RMR) is calculated by plotting energy expenditure against radar output, that are both averaged over 30-min periods. The intercept of the regression line at the lowest radar output represents the energy expenditure in the inactive state (= RMR), which consists of SMR and DIT (31). DIT is determined by subtracting SMR from RMR. AEE is determined by subtracting SMR and DIT from 24-h energy expenditure.

6.3.3. Substrate oxidation

Carbohydrate, fat, and protein oxidation are calculated from the measurements of oxygen consumption, carbon dioxide production, and urinary nitrogen excretion by using the formula of Carpenter in Brouwer et al (32). Urine samples are collected from the second void on the day subjects enter the respiration chamber to 20:30h on the next day. Samples, 3 per 36 hrs,

in order to determine the circadian rhythm of substrate oxidation are collected in containers with 10 ml HCl to prevent nitrogen loss through evaporation. Volume and nitrogen concentration are measured, the latter with a nitrogen analyzer (CHN-O-Rapid; Heraeus, Hanau, Germany). Urinary nitrogen needs to be collected in order to be able to calculate the RQ and protein balance correctly.

6.3.4. Appetite profile

Appetite profile will be measured using anchored 100-mm VAS. During each respiration chamber session these questionnaires will be completed every waking hour, and before and after every meal on feelings of hunger, fullness, appetite, satiety, thirst, prospective food consumption, and desire to eat. The scale is anchored from ‘not at all’ on the left to ‘extremely’ on the right.

6.3.5. Mood

The POMS questionnaire contains 70 adjectives that are rated on a five-point scale, anchored by ‘much like this’ to ‘much unlike this’ and is divided into five subscales (depression, tension, confusion, fatigue and anger), each scoring a maximum of 35. An increase in POMS scores is associated with a worsening mood, except in the case of ‘vigor’. The STAI state questionnaire refers to the transitory emotional response involving unpleasant feelings of tension and apprehensive thoughts. The STAI scale is composed of 20 questions rated on a four-point scale, ranging from ‘much like this’ to ‘much unlike this’ and requires that subjects describe how they feel generally (Trait Anxiety Inventory scale), or at a specific moment (State Anxiety Inventory scale). The questionnaire can score a maximum of 80, whereby increased scores are associated with an increase in anxiety. Completing both questionnaires will take about 3 minutes.

6.3.6. Energy intake and food choice

Subjects will be fed in energy balance during three days before the test sessions. The energy content of this diet will be based on basal metabolic rate (BMR), individually calculated with the equation of Harris-Benedict (33), and multiplied by a physical activity level (PAL) of 1.7. In the respiration chamber energy requirements will be calculated based on a PAL of 1.35. Subject will be fed 100% of their daily energy requirements in the conditions ‘100%Control’ and ‘100%CAPS’, and will receive 75% of their daily energy requirements in the condition ‘75%Control’ and ‘75%CAPS’. Energy intake will be divided over the meals as 20% for breakfast (08:30h), 40% for lunch (13:30h), and 40% for dinner (18:30h). Subjects have to completely finish all drinks and meals within 30 minutes, except for the food provided at dinner on the second day. Each food item provided for dinner on the second day will be

weighted before it is offered to the subjects. After 30 minutes the left food items will be weighted again, whereafter the amount or each food item consumed will be calculated. Capsaicin will be given at a dose of 80.000 SHU, corresponding to an amount of 5.12 mg capsaicin (2.05 g of red pepper), with every meal. The given dose of each component will not exceed the recommended daily dose. Subjects will be allowed to drink water, tea and decaffeinated coffee *ad libitum* during their stay in the respiration chamber.

All food items will be bought in a normal Dutch supermarket. The meals will be freshly prepared by a dietician in a research kitchen, just before they are offered to the subjects. Meal composition, calculated for a representative men (age 25 y, weight 75 kg, height 1.80 m; Table 1,2).

Table 1. Diet to be consumed at home

**Food item**	**Amount (g)**
Breakfast	
Wholegrain bread	107
Low-fat margarine	12
Strawberry jam	30
Liver paste	23
Chocolate milk	230
Lunch	
Wholegrain bread	134
Low-fat margarine	15
Meatloaf	33
Liver paste	28
Ginger cake	41
Milk	338
Apple	218
Raisin biscuits	58
Diner	
Kale with sausage and jus	691
Fruit cocktail	329
Vanilla custard	227
Orange juice	395

Table 2. Diet to be consumed during test

Food item	**Amount (g)**
Breakfast	
Tomato juice	250
Wholegrain bread	73
Low-fat margarine	9
Strawberry jam	18
Liver paste	13
Chocolate milk	197
Lunch	
Tomato juice	250
Wholegrain bread	118
Low-fat margarine	14
Meatloaf	29
Liver paste	22
Ginger cake	32
Low-fat mild	197
Apple	168
Raisin biscuits	45
Diner	
Tomato juice	250
Vegetable lasagne	592
Mixed salad	99
Dressing	27
Low-fat yoghurt	237
Orange juice	197

6.4. Withdrawal of individual subjects

Subjects can leave the study at any time for any reason if they wish to do so without any consequences. The investigator can decide to withdraw a subject from the study for urgent medical reasons.

6.5. Replacement of individual subjects after withdrawal

New subjects will replace subjects that are withdrawed from the study.

6.6. Follow-up of subjects withdrawn from treatment

Subjects withdrawn from treatment will not be followed-up after eventually handing over responsibilities to medical personnel.

7. SAFETY REPORTING

7.1. Section 10 WMO event

In accordance to section 10, subsection 1, of the WMO, the investigator will inform the subjects and the reviewing accredited METC if anything occurs, on the basis of which it appears that the disadvantages of participation may be significantly greater than was foreseen in the research proposal. The study will be suspended pending further review by the accredited METC, except insofar as suspension would jeopardise the subjects' health. The investigator will take care that all subjects are kept informed.

7.2. Adverse and serious adverse events

Adverse events are defined as any undesirable experience occurring to a subject during the study, whether or not considered related to the experimental intervention. All adverse events reported spontaneously by the subject or observed by the investigator or his staff will be recorded.

A serious adverse event (SAE) is any untoward medical occurrence or effect that at any dose:

- results in death;
- is life threatening (at the time of the event);
- requires hospitalisation or prolongation of existing inpatients' hospitalisation;
- results in persistent or significant disability or incapacity;
- is a congenital anomaly or birth defect;
- is a new event of the trial likely to affect the safety of the subjects, such as an unexpected outcome of an adverse reaction, lack of efficacy of an IMP used for the treatment of a life threatening disease, major safety finding from a newly completed animal study, etc.

All SAEs will be reported through the web portal *ToetsingOnline* to the accredited METC that approved the protocol, within 15 days after the sponsor has first knowledge of the serious adverse reactions.
SAEs that result in death or are life threatening should be reported expedited. The expedited reporting will occur not later than 7 days after the responsible investigator has first knowledge of the adverse reaction. This is for a preliminary report with another 8 days for completion of the report.

7.3. Follow-up of adverse events

All adverse events will be followed until they have abated, or until a stable situation has been reached. Depending on the event, follow up may require additional tests or medical procedures as indicated, and/or referral to the general physician or a medical specialist.

8. STATISTICAL ANALYSIS

8.1. Descriptive statistics

Data will be presented as means ± standard error of the mean (SEM). Energy expenditure will be expressed as total 36h energy expenditure, as well as for the different components (SMR, DIT and AEE). Substrate oxidation will be calculated as carbohydrate, fat and protein oxidation and as RQ. Total energy and macronutrient intake will be calculated to subsequently calculate energy and macronutrient balance. VAS ratings and POMS and STAI scores will be described. Area under the curve (AUC) and area above the curve (AAC) will be calculated using the trapezoidal method for all the variables described above.

8.2. Univariate analysis

The Statistical Package for the Social Sciences (SPSS) will be used to perform univariate analyses. ANOVA repeated measures will be performed to determine possible differences in energy expenditure and its components, substrate oxidation, *ad libitum* energy intake, energy and macronutrient balance, appetite profiles and mood scores between the four conditions. Multiple linear regression analyses will be performed to determine the independent contribution of gender, age, and factors describing body composition and body fat distribution on the dependent variables. All statistical tests will be two-sided and differences will be considered statistical significant if $p<.05$.

9. ETHICAL CONSIDERATIONS

9.1. Regulation statement

The study will be conducted according to the principles of the Declaration of Helsinki (9th version, October 2008, Seoul) and in accordance with the Dutch version of the Medical Research Involving Human Subjects Act (in Dutch: Wet Medisch-wetenschappelijk Onderzoek met mensen; WMO).

9.2. Recruitment and consent

Subjects from the student population will be recruited by advertisements on notice boards at the Maastricht University. They will be informed about the study by reading the provided written ‘subject information’. The researchers will orally check their comprehensiveness. An independent person who’s well informed about the study could eventually provide additional information, and subjects will be referred to the CCMO brochure ‘General information for research participants’ that contains general information about medical-scientific research. After being well-informed subjects will get one week before they have to decide to participate in the study. All subjects will confirm their approval for participation by signing an informed consent form.

9.3. Benefits and risks assessment, group relatedness

This study does not have any benefits for the subjects themselves, but will give possible new knowledge for treatment of obesity. Besides, the study does not include any major risk for the subjects. Anthropometric and body composition measurements, performed within several minutes during the screening, will not be invasive for the subjects. Deuterium dilution has been shown to be a safe method for determining total body water. Subjects have to consume an amount of about 150 mL of water in which a small amount of deuterium is diluted. The taste of the solution is comparable to normal water (28). The completion of screening questionnaires will take about 30 minutes.
Accidential findings during the screening related to health status will be communicated to the concerned subjects, whereafter they can individually undertake further steps.

Although the space in the comfortable designed respiration chamber is limited, it is still possible to move freely. Curtains ensure privacy when needed. The door of the respiration chamber is closed, but in case of emergency it could always be opened from inside to leave the chamber. Safety precautions include a fire alarm and extinguisher, emergency power and lighting, subject registration on a building fire-alert panel and short access to a safe escape route. The registration of oxygen consumption and carbon dioxide production will be performed during an unconscious process. The air in the chamber is continuously regulated.

Besides filling in the questionnaires hourly (which takes about 3 minutes every hour), urine collection over the day and prescribed meal and sleep times, subjects can freely spend their time in the respiration chamber.

Urine sampling will be done in 2L urine bottles added with 10 mL of diluted HCl, which might pose a risk for the subjects. Subjects will be carefully instructed how to handle the bottles to reduce these risks.

Further, there are no risks for the subject in consuming any of the provided meals, because people with certain food allergies are excluded for participation and all meals are composed of regular food items available in normal Dutch supermarkets. The addition of capsaicin (McCormick; origin, India; sample ID EC88746100) to the meals will not form any health risk. Capsaicin is a natural product, which is safe in the given dose that will not exceed the maximum recommended daily dose. The dosage used based upon the maximal dosage given in previous studies (8, 19, 20). The same specific form of red pepper has previously been used in a study of Ludy et al, investigating the effects of red pepper on thermogenesis and appetite (11).
All food items, including capsaicin, are suitable for humans. The commercial available food products will be bought by the researchers and immediately after purchase transported by car to the University. Refrigerated products will be taken in deepfreeze bags. After arrival at the University the refrigerated products will be stored immediately at either a 4°C fridge or a -20°C freezer in the research kitchen from the Department of Human Biology. This research kitchen meets all the criteria for safe and proper use of food items in human clinical studies. Capsaicin will be processed, handled, packaged and transported by McCormick, as described in the HACCP-analysis. After arrival at the University the product will be stored in a locked food depot in the research kitchen. Capsaicin will be stored for a maximum period of one year, which corresponds to the maximum duration of the study and will not exceed the expiration date of the product. A certified dietician controls the storage of the products. Before preparation of the meals the dietician will check the expiration dates of the food items. Expired food items will immediately be removed. All food items will be prepared in the research kitchen from the Department of Human Biology by a certified dietician just before they are offered to the subjects. Fresh food items will be used every day, and food items will be stored for a maximum period of one week. Capsaicin will be weighed and mixed with the tomato juice by the dietician just before it is offered to the subjects. Addition of capsaicin will not largely influence the taste of the tomato juice, although the juice will be slightly spicier. Further information can be found in the guideline 'MEC azM/UM for preparation, delivery and administration of products for human usage in clinical studies'.

9.4. Compensation for injury

The investigator has a liability insurance, which is in accordance with article 7, subsection 6 of the WMO.

The sponsor also has an insurance which, is in accordance with the legal requirements in the Netherlands (Article 7 WMO and the Measure regarding Compulsory Insurance for Clinical Research in Humans of 23th June 2003). This insurance provides cover for damage to research subjects through injury or death caused by the study.

€ 450.000,-- (i.e. four hundred and fifty thousand Euro) for death or injury for each subject who participates in the Research;

€ 3.500.000,-- (i.e. three million five hundred thousand Euro) for death or injury for all subjects who participate in the Research;

€ 5.000.000,-- (i.e. five million Euro) for the total damage incurred by the organisation for all damage disclosed by scientific research for the Sponsor as 'verrichter' in the meaning of said Act in each year of insurance coverage.

The insurance applies to the damage that becomes apparent during the study or within 4 years after the end of the study.

9.5. Incentives

Per test session subjects will receive a financial compensation of 75 euro, which is 300 euro in total after completion of the study. Possible travel expenses will be declared.

10. ADMINISTRATIVE ASPECTS AND PUBLICATION

10.1. Handling and storage of data and documents

Subjects' personal information will be confidentially handled during the study, according to the Dutch Personal Data Protection Act (Wet bescherming persoonsgegevens; Wbp). Results will be linked to the intervention and not to individual subjects. Subject numbers (e.g. number 1) in combination with treatment codes (100%control, code A; 75%Control, code B, 100%CAPS, code C; 75%CAPS, code D) will be used throughout the study. Only the researcher is familiar with subject information, so privacy of personal information is guaranteed. If desirable, subjects will be informed about their personal study results at the end of the study. Collected body material (urine) will be destroyed after publication of the results. Personal data will be kept for a period of 15 year, which is the legal time to keep medical data. Hereafter the data will be destroyed.

10.2. Amendments

Amendments are changes made to the research after a favourable opinion by the accredited METC has been given. All amendments will be notified to the METC that gave a favourable opinion.

10.3. Annual progress report

The investigator will submit a summary of the progress of the trial to the accredited METC once a year. Information will be provided on the date of inclusion of the first subject, numbers of subjects included and numbers of subjects that have completed the trial, serious adverse events/ serious adverse reactions, other problems, and amendments.

10.4. End of study report

The investigator will notify the accredited METC of the end of the study within a period of 8 weeks. The end of the study is defined as the last patient's last visit.

In case the study is ended prematurely, the investigator will notify the accredited METC, including the reasons for the premature termination.

Within one year after the end of the study, the investigator will submit a final study report with the results of the study, including any publications/abstracts of the study, to the accredited METC.

10.5. Public disclosure and publication policy

The 'CCMO Statement on publication policy' will be followed to publish research results after the end of the study.

11. REFERENCES

1. Clinical Guidelines on the Identification, Evaluation, and Treatment of Overweight and Obesity in Adults--The Evidence Report. National Institutes of Health. Obesity research. 1998 Sep;6 Suppl 2:51S-209S.
2. Aucott LS. Influences of weight loss on long-term diabetes outcomes. The Proceedings of the Nutrition Society. 2008 Feb;67(1):54-9.
3. Repas TB. Challenges and strategies in managing cardiometabolic risk. The Journal of the American Osteopathic Association. 2007 Apr;107(4 Suppl 2):S4-11.
4. Kaplan LM. Body weight regulation and obesity. J Gastrointest Surg. 2003 May-Jun;7(4):443-51.
5. Kurpad AV, Muthayya S, Vaz M. Consequences of inadequate food energy and negative energy balance in humans. Public health nutrition. 2005 Oct;8(7A):1053-76.
6. Yoshioka M, St-Pierre S, Suzuki M, Tremblay A. Effects of red pepper added to high-fat and high-carbohydrate meals on energy metabolism and substrate utilization in Japanese women. The British journal of nutrition. 1998 Dec;80(6):503-10.
7. Yoshioka M, Doucet E, Drapeau V, Dionne I, Tremblay A. Combined effects of red pepper and caffeine consumption on 24 h energy balance in subjects given free access to foods. The British journal of nutrition. 2001 Feb;85(2):203-11.
8. Lejeune MP, Kovacs EM, Westerterp-Plantenga MS. Effect of capsaicin on substrate oxidation and weight maintenance after modest body-weight loss in human subjects. The British journal of nutrition. 2003 Sep;90(3):651-59.
9. Diepvens K, Westerterp KR, Westerterp-Plantenga MS. Obesity and thermogenesis related to the consumption of caffeine, ephedrine, capsaicin, and green tea. American journal of physiology. 2007 Jan;292(1):R77-85.
10. Westerterp-Plantenga M, Diepvens K, Joosen AM, Berube-Parent S, Tremblay A. Metabolic effects of spices, teas, and caffeine. Physiology & behavior. 2006 Aug 30;89(1):85-91.
11. Ludy MJ, Mattes RD. The effects of hedonically acceptable red pepper doses on thermogenesis and appetite. Physiology & behavior. 2011 Mar 1;102(3-4):251-8.
12. Kawada T, Sakabe S, Watanabe T, Yamamoto M, Iwai K. Some pungent principles of spices cause the adrenal medulla to secrete catecholamine in anesthetized rats. Proceedings of the Society for Experimental Biology and Medicine Society for Experimental Biology and Medicine (New York, NY. 1988 Jun;188(2):229-33.
13. Kawada T, Watanabe T, Takaishi T, Tanaka T, Iwai K. Capsaicin-induced beta-adrenergic action on energy metabolism in rats: influence of capsaicin on oxygen consumption, the respiratory quotient, and substrate utilization. Proceedings of the Society for Experimental Biology and Medicine Society for Experimental Biology and Medicine (New York, NY. 1986 Nov;183(2):250-6.

14. Watanabe T, Kawada T, Kurosawa M, Sato A, Iwai K. Adrenal sympathetic efferent nerve and catecholamine secretion excitation caused by capsaicin in rats. The American journal of physiology. 1988 Jul;255(1 Pt 1):E23-7.
15. Watanabe T, Kawada T, Yamamoto M, Iwai K. Capsaicin, a pungent principle of hot red pepper, evokes catecholamine secretion from the adrenal medulla of anesthetized rats. Biochemical and biophysical research communications. 1987 Jan 15;142(1):259-64.
16. Eldershaw TP, Colquhoun EQ, Bennett KL, Dora KA, Clark MG. Resiniferatoxin and piperine: capsaicin-like stimulators of oxygen uptake in the perfused rat hindlimb. Life sciences. 1994;55(5):389-97.
17. Caterina MJ, Leffler A, Malmberg AB, Martin WJ, Trafton J, Petersen-Zeitz KR, et al. Impaired nociception and pain sensation in mice lacking the capsaicin receptor. Science (New York, NY. 2000 Apr 14;288(5464):306-13.
18. Yoshioka M, St-Pierre S, Drapeau V, Dionne I, Doucet E, Suzuki M, et al. Effects of red pepper on appetite and energy intake. The British journal of nutrition. 1999 Aug;82(2):115-23.
19. Westerterp-Plantenga MS, Smeets A, Lejeune MP. Sensory and gastrointestinal satiety effects of capsaicin on food intake. International journal of obesity (2005). 2005 Jun;29(6):682-8.
20. Smeets AJ, Westerterp-Plantenga MS. The acute effects of a lunch containing capsaicin on energy and substrate utilisation, hormones, and satiety. European journal of nutrition. 2009 Jun;48(4):229-34.
21. Reinbach HC, Smeets A, Martinussen T, Moller P, Westerterp-Plantenga MS. Effects of capsaicin, green tea and CH-19 sweet pepper on appetite and energy intake in humans in negative and positive energy balance. Clinical nutrition (Edinburgh, Scotland). 2009 Jun;28(3):260-5.
22. Albrecht RR, Ewing SJ. Standardizing the administration of the Profile of Mood States (POMS): development of alternative word lists. Journal of personality assessment. 1989 Spring;53(1):31-9.
23. Tenenbaum G, Furst D, Weingarten G. A statistical reevaluation of the STAI anxiety questionnaire. Journal of clinical psychology. 1985 Mar;41(2):239-44.
24. Tremblay A. Differences in fat balance underlying obesity. Int J Obes Relat Metab Disord. 1995 Dec;19 Suppl 7:S10-S4; discussion S5-6.
25. Hochstenbach-Waelen A, Veldhorst MA, Nieuwenhuizen AG, Westerterp-Plantenga MS, Westerterp KR. Comparison of 2 diets with either 25% or 10% of energy as casein on energy expenditure, substrate balance, and appetite profile. The American journal of clinical nutrition. 2009 Mar;89(3):831-8.

26. Hochstenbach-Waelen A, Westerterp-Plantenga MS, Veldhorst MA, Westerterp KR. Single-protein casein and gelatin diets affect energy expenditure similarly but substrate balance and appetite differently in adults. J Nutr. 2009 Dec;139(12):2285-92.
27. Yoshioka M, Imanaga M, Ueyama H, Yamane M, Kubo Y, Boivin A, et al. Maximum tolerable dose of red pepper decreases fat intake independently of spicy sensation in the mouth. The British journal of nutrition. 2004 Jun;91(6):991-5.
28. van Marken Lichtenbelt WD, Westerterp KR, Wouters L. Deuterium dilution as a method for determining total body water: effect of test protocol and sampling time. The British journal of nutrition. 1994 Oct;72(4):491-7.
29. Schoeller DA, van Santen E, Peterson DW, Dietz W, Jaspan J, Klein PD. Total body water measurement in humans with 18O and 2H labeled water. The American journal of clinical nutrition. 1980 Dec;33(12):2686-93.
30. Westerterp KR, Wouters L, Van Marken Lichtenbelt WD. The Maastricht protocol for the measurement of body composition and energy expenditure with labeled water. Obes Res. 1995;3S1:49-57.
31. Schoffelen PF, Westerterp KR, Saris WH, Ten Hoor F. A dual-respiration chamber system with automated calibration. J Appl Physiol. 1997 Dec;83(6):2064-72.
32. Brouwer E. On simple formulae for calculating the heat expenditure and the quantities of carbohydrate and fat oxidized in metabolism of men and animals, from gaseous exchange (Oxygen intake and carbonic acid output) and urine-N. Acta physiologica et pharmacologica Neerlandica. 1957;6:795-802.
33. Harris JA, Benedict FG. A Biometric Study of Human Basal Metabolism. Proceedings of the National Academy of Sciences of the United States of America. 1918 Dec;4(12):370-3.
